# Traditional Chinese medicine for premature ventricular contraction caused by obstructive sleep apnea: A case report and literature review

**DOI:** 10.1097/MD.0000000000041206

**Published:** 2025-01-31

**Authors:** Qing-Qing Yue, Yi-Fang Hao, Ting Li, Han-Ying Miao, Bing-Chang Chen, Sheng-Yao Li

**Affiliations:** a National Clinical Research Center for Chinese Medicine Cardiology, Xiyuan Hospital, China Academy of Chinese Medical Sciences, Beijing, China; b Shanxi University of Chinese Medicine, Taiyuan, China; c Shanxi Hospital of Xiyuan Hospital, China Academy of Chinese Medical Sciences, Taiyuan, China.

**Keywords:** case report, Huanglian Wendan Decoction, obstructive sleep apnea, premature ventricular contraction, traditional Chinese medicine

## Abstract

**Rationale:**

Premature ventricular contraction (PVC) is a common type of arrhythmia, and obstructive sleep apnea (OSA) is a common trigger for this condition. Some patients still have PVC, even if ventilation is improved by wearing a respirator. Traditional Chinese medicine (TCM) has a long history of arrhythmia treatment, and this is the first report of a patient with PVC caused by OSA treated with TCM. Twenty-four-hour Holter showed that the number of PVCs decreased from 8968 to 0 before and after TCM treatment, and discomfort symptoms disappeared completely.

**Patient concerns:**

A 37-year-old middle-aged man, with no history of hypertension, diabetes, hyperlipidemia, or other underlying diseases, had been suffering from OSA for over 1 year and currently uses a respirator to sleep. He was diagnosed with PVC at Beijing Anzhen Hospital 1 year ago and had been taking propafenone hydrochloride tablets following the physician’s advice, but palpitation had not been relieved. The patient did not consider surgery and hoped to take TCM to treat the palpitations.

**Diagnoses:**

The patient was diagnosed with PVC. After excluding other factors that could cause PVC, it was ultimately considered that the patient’s PVC was related to OSA.

**Interventions:**

The patient visited Xiyuan Hospital on December 5, 2023, on the basis of existing Western medicine and TCM, using the modified Huanglian Wendan Decoction.

**Outcomes:**

After taking TCM for 21 days, the Holter tests were conducted again. The results showed that the number of PVCs decreased from 8968 to 0 within 24 hours. Meanwhile, his palpitations were relieved, and the dosage of propafenone hydrochloride tablets was halved. After 7 days, he did not experience any discomfort; therefore, propafenone hydrochloride tablets were discontinued and TCM was exclusively administered. He received 7 additional courses in TCM. During the final consultation on April 23, 2024, the patient reported no discomfort, and snoring improved.

**Lessons:**

After treatment with TCM, the patient’s palpitations disappeared completely and snoring improved, which proved that TCM can treat PVC caused by OSA. To verify this conclusion, more high-quality research is necessary to establish the efficacy and underlying mechanisms of TCM in treating PVC caused by OSA.

## 1. Introduction

Premature ventricular contraction (PVC) occurs when an ectopic rhythm point in any part of the ventricle or the interventricular septum emits an electrical impulse before the sinus node impulse reaches the ventricles, causing ventricular depolarization. PVC is a common arrhythmia, with causes including organic heart disease (such as cardiomyopathy and coronary heart disease), non-cardiac diseases (such as hyperthyroidism and obstructive sleep apnea [OSA]), medications (such as cardiac glycosides and anesthetics), and emotional factors. Generally, patients with PVC who have no symptoms or only mild symptoms do not require medication. They only need to avoid triggers such as smoking, coffee, and stress. When symptoms are more severe, antiarrhythmic drugs such as beta blockers, non-dihydropyridine calcium channel blockers, and amiodarone can be selected. Catheter ablation is another treatment method; however, except for a small number of frequent PVC originating from the right ventricular outflow tract or the left ventricular posterior septum, the success rate of PVC originating from other sites is relatively low, the postoperative recurrence rate is high, and many patients refuse to undergo catheter ablation. Therefore, the majority of patients with PVC tend to take medication, but drug therapy has many side effects, such as decreased in cardiac contractility, suppression of cardiac conduction, and induction of new arrhythmia. Furthermore, treatment efficacy varies significantly among individuals.

The incidence of OSA is gradually increasing, ranging from 2% to 4% in developed countries such as Europe and the United States,^[1]^ and as high as 11% in China.^[2]^ Gender, age, obesity, anatomical abnormalities of the upper airway, genetic factors, and neuromuscular diseases are risk factors for OSA. Weight loss and continuous positive airway pressure (CPAP) are recognized as effective treatment methods, with CPAP being the primary treatment for patients with moderate-to-severe OSA.^[3]^

OSA is a sleep-related breathing disorder characterized by snoring with apnea during sleep and excessive daytime sleepiness as its main clinical manifestations. OSA is a source of various chronic diseases and is closely associated with cardiovascular diseases.^[4]^ A study has found that patients with OSA have a significantly increased incidence of arrhythmia during sleep.^[5]^ Even in patients with mild OSA, there is an association between OSA and arrhythmia, with an increased prevalence of PVC in middle-aged patients primarily with mild or moderate OSA.^[6]^ The fundamental mechanism by which OSA induces PVC remains unclear; however, OSA is usually accompanied by hypoxemia and hypercapnia, which can affect the cardiovascular and autonomic nervous systems. These mechanisms are related to dysfunction of the autonomic nervous system,^[7]^ intra-thoracic pressure,^[8]^ myocardial ischemia,^[9]^ oxidative stress,^[10]^ inflammatory response,^[11]^ and disruption of sleep structure. Increased sympathetic nervous tension leads to an increased release of catecholamines in myocardial tissue, which causes tachycardia and increases blood pressure and myocardial oxygen demand. If this process fails to compensate, myocardial ischemia may ensue, subsequently leading to arrhythmia. In contrast, intermittent hypoxemia and hypercapnia affect myocardial electrical stability, which triggers arrhythmia.^[12]^ When these immediate physiological stresses occur, continuously, changes in biological effects, ultimately affects cardiac structure and electrophysiology, and increase the risk of arrhythmia.^[13]^ Current research suggests that CPAP improves OSA symptoms^[14]^ and reduces the incidence of arrhythmia,^[15]^ but the improvement in PVC caused by OSA remains to be confirmed.

Evidence-based practices have accumulated in Traditional Chinese medicine (TCM) for the treatment of PVC. For example, the Chinese patent medicines Shen Song Yang Xin capsules^[16]^ and Wen Xin granules,^[17]^ acupuncture therapy,^[18]^ and Ba Duan Jin,^[19]^ all of which have shown satisfactory effects in treating PVC. Chinese herbal medicine, an important component of TCM culture, has been confirmed in multiple studies for its effectiveness in treating PVC.^[20]^ However, there have been no reported cases of TCM for OSA causing PVC and reducing the number of PVC episodes to zero. This case is the first to demonstrate the efficacy and safety of TCM in treating PVC caused by OSA. In the future, TCM may become the preferred strategy for treating PVC caused by OSA.

## 2. Case description

On December 5, 2023, a 37-year-old middle-aged man presented with palpitation for 6 months as the main symptom. The patient had no history of hypertension, coronary heart disease, diabetes, or other underlying diseases; no family history of cardiovascular disease; and no history of smoking or alcohol use. His current symptoms included palpitations, easy fatigue, indigestion, acid reflux, and snoring during sleep, requiring the use of a respirator.

**Figure 1.**
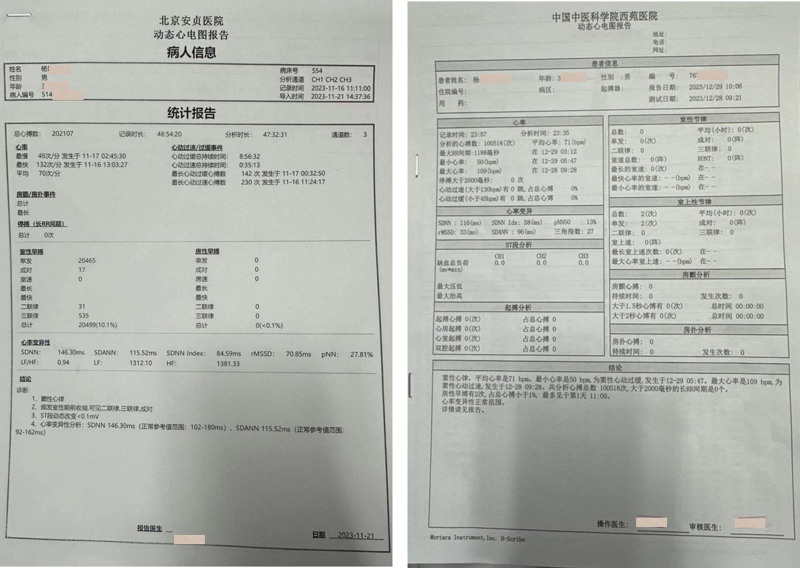
The left and right show the Holter before TCM and after TCM. TCM = traditional Chinese medicine.

In 2023, he was diagnosed with OSA at Peking University People’s Hospital and had been using a respirator to sleep ever since. One year ago, the patient underwent a routine physical examination, and the report showed that there was no abnormality in thyroid function, electrolytes, blood routine, liver function, renal function, etc; however, electrocardiogram revealed PVC. There were no symptoms at the time; therefore, the patient did not pay much attention to it. One month after the physical examination, the patient began to feel palpitations, and the symptoms gradually worsened. On November 21, 2023, the patient underwent a comprehensive examination at Beijing Anzhen Hospital, including coronary computed tomographic angiography, echocardiography, and Holter (Fig. 1–Fig. 3) and so on. He was ultimately diagnosed with PVC, and instructed to take propafenone hydrochloride tablets orally, 3 times a day, 150 mg each time, and recommended that the patient be treated for PVC through catheter ablation. The patient refused the suggestion and only accepted propafenone hydrochloride tablets on time. The patient showed acid reflux after taking propafenone hydrochloride tablets, and palpitations did not improve.

**Figure 2.**
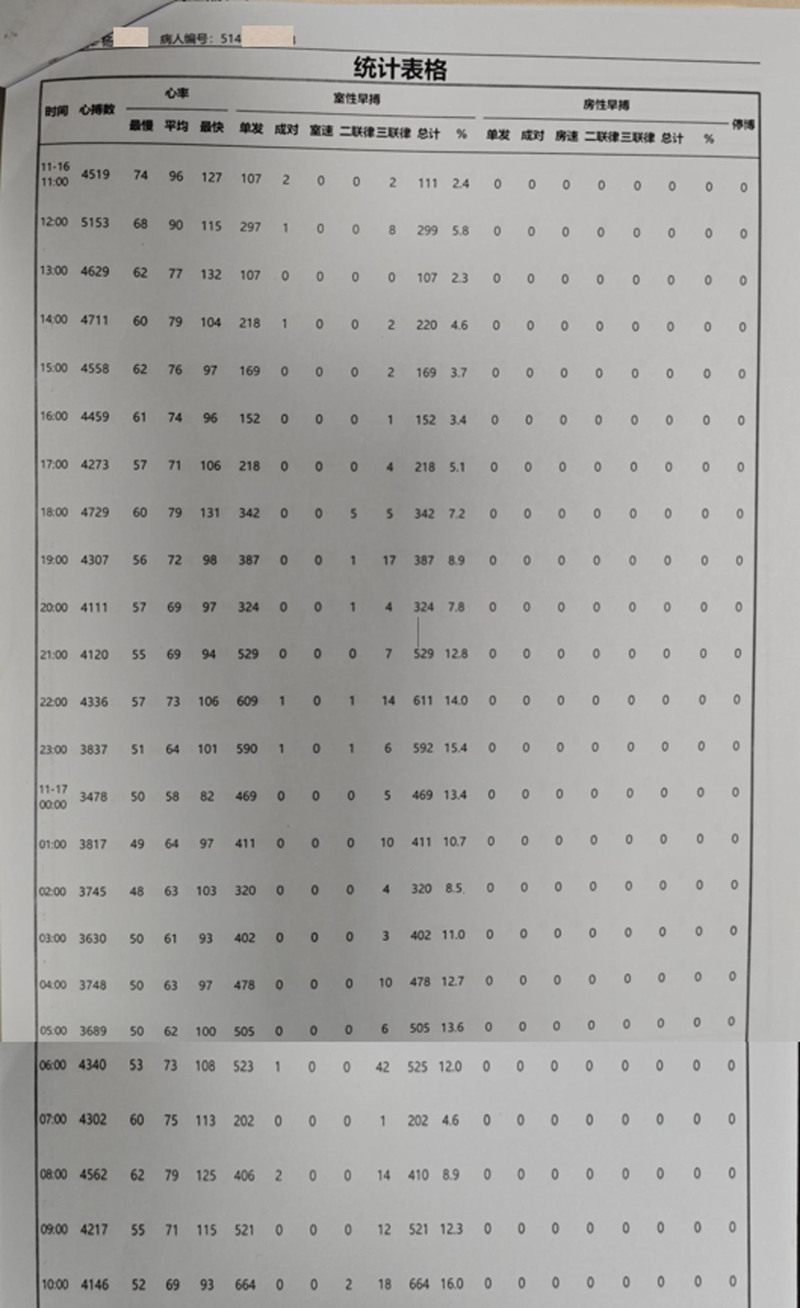
The first 24 hours of the Holter before TCM. TCM = traditional Chinese medicine.

**Figure 3.**
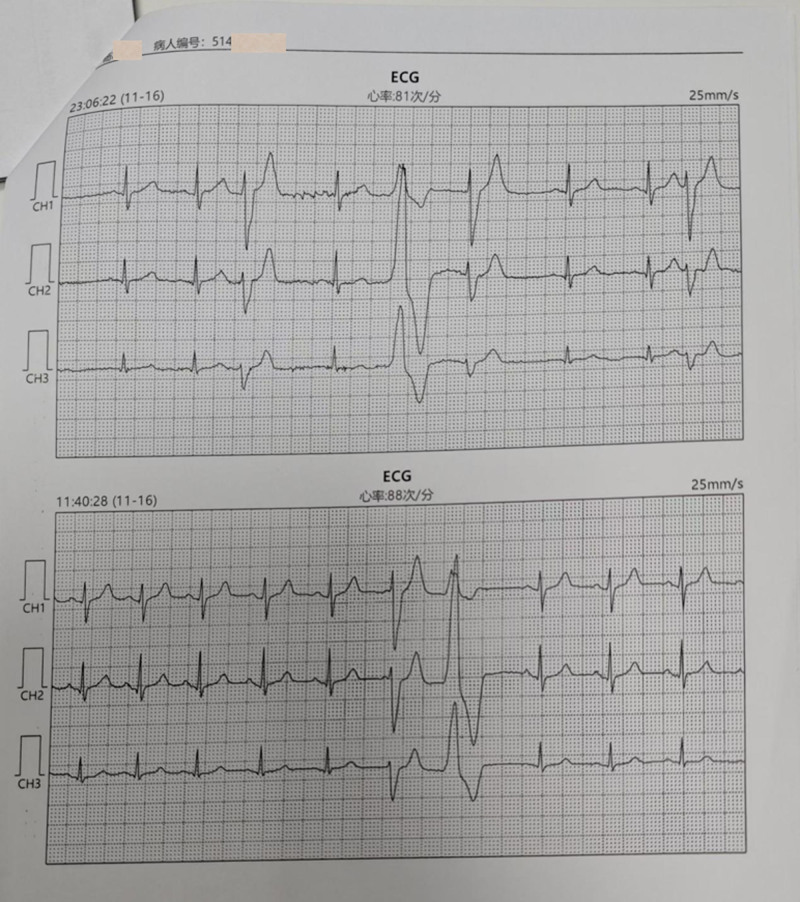
Surface ECG with PVC. (The ECG on the top was at 11:06 pm, and the other 1 was at 11:40 am). ECG = electrocardiogram, PVC = premature ventricular contraction.

On December 5, 2023, the patient visited Xiyuan Hospital, hoping to alleviate palpitations through TCM. Analysis of the patient: the Holter monitor showed PVC, while coronary computed tomographic angiography and echocardiography did not reveal any significant abnormalities, which eliminated organic heart disease. Further analysis of the causes of the PVC was performed. The patient’s thyroid function, electrolytes, and other potential causes of PVC were examined, all of which were normal. The patient was emotionally stable and denied taking other medications. By ruling out other common causes of PVC, combined with the Holter monitoring, a significant increase in the number of PVCs during sleep (Fig. 2), and the patient’s ineffective response to antiarrhythmic medication were observed. Therefore, PVC was considered that his PVC was related to OSA.

**Figure 4.**
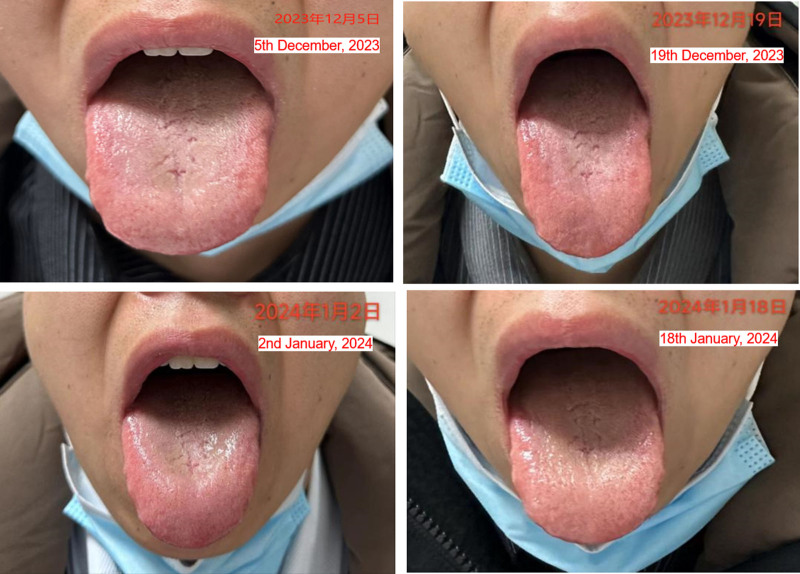
Tongue image of the patient during the first 4 courses of treatment.

Analyzed the patient from the perspective of TCM: the tongue surface presented yellow color (Fig. 4), with a small blister on the tip, which indicated the presence of fire evil in heart. Additionally, the patient’s tongue showed significant tooth marks on both sides; he reported a bitter taste in the mouth and his pulse was slippery. From TCM perspective, the reason why the patient experienced discomfort symptoms was because phlegm and heat, the 2 types of evils disturbed heart. Considering the patient’s present condition, the modified Huanglian Wendan Decoction was selected. It consisted of a total of 16 kinds of Chinese herbs, which were boiled in water for about 30 minutes and then consumed once after breakfast and dinner, for 14 days.

On December 19, 2023, the patient returned for a second consultation, reporting that there was not much improvement in symptoms during the first treatment course. However, the small blister on the tongue disappeared, which was considered a sign that phlegm and heat gradually receded.

On January 2, 2024, the patient returned for a third consultation and reported a significant improvement in palpitations during the second treatment course. On December 29, 2023, Holter was performed again, and the results showed that the number of PVC was 0 (Fig. 1). Thus, propafenone hydrochloride tablets were discontinued. At that time, his tongue showed significant saliva production, which indicated that the evils gradually disappeared, and the condition gradually improved. The patient’s symptoms and examination results proved that TCM was effective; therefore, he continued taking the second course of TCM for 14 days.

On January 18, 2024, during the fourth consultation, the patient reported no discomfort, other than thirst. Although saliva was presented on the tongue, it appeared rough, which may be due to the excessive use of heat-clearing and phlegm-resolving herbs that harm the body fluid, causing the tongue to become dry. Therefore, some herbs with the function of supplementing fluids were added to the TCM.

During the fourth treatment course, the patient experienced a cold and returned for consultation on January 24, 2024. The TCM prescription was readjusted based on the cold symptoms. This treatment course lasted for 7 days, primarily targeting cold symptoms, and after 7 days, the patient’s cold recovered. Subsequently, the patient insisted on coming for a consultation every 14 days to take TCM and reported that the palpitation had not recurred and snoring had also improved. In the following 7 treatment courses, the patient’s symptoms changed little; therefore, each course of TCM only increased or decreased 1 or 2 herbs. Therefore, only specific prescriptions for the first 4 treatment courses are listed (Table 1).

**Table 1:** Four courses of Chinese herbs used by the patient between December 5, 2023, and January 24, 2024.

Chinese herbs	First course of treatment December 5, 2023–December 18, 2023	Second course of treatment December 19, 2023–January 1, 2024	Third course of treatment January 2, 2024–January 17, 2024	Fourth course of treatment January 18, 2014–January 24, 2024
Coptis	10 g	15 g	15 g	15 g
Immature bitter orange	15 g	15 g	15 g	15 g
Caulis bambusae in taeniam	10 g	10 g	10 g	10 g
Dried orange peel	15 g	15 g	15 g	15 g
Rhizoma pinelliae preparata	9 g	9 g	9 g	9 g
Fresh ginger	10 g	10 g	10 g	10 g
Agrimonia pilosa	30 g	30 g	30 g	60 g
Raw oyster shell	30 g	30 g	30 g	–
Rhizoma nerdostachyos	20 g	20 g	20 g	20 g
Acorus gramineus	15 g	15 g	15 g	15 g
Radix curcumae	15 g	15 g	15 g	20 g
Poria cocos	20 g	30 g	30 g	–
Astragalus	30 g	30 g	30 g	30 g
Rhizoma corydalis	15 g	15 g	15 g	15 g
Codonopsis root	30 g	30 g	30 g	30 g
Jujube A	30 g	30 g	30 g	30 g
Husked sorghum	–	30 g	30 g	–
Tuber fleece flower stem	–	30 g	30 g	30 g
Polygala tenuifolia	–	–	–	10 g
Rhizoma anemarrhenae	–	–	–	10 g
Poria with hostwood	–	–	–	20 g
Calcined oyster shell	–	–	–	30 g

The patient’s last consultation was on April 23, 2024, and he said that he was feeling fine with no discomfort.

## 3. Discussion

TCM has achieved significant results in OSA treatment. Zhou^[21]^ used the Zhi Han Decoction to treat patients with OSA and found that the overall effective rate of symptom improvement was as high as 82.5%. There were significant improvements in the lowest nocturnal blood oxygen saturation and apnea hypopnea index after treatment. Yin^[22]^ used the Chinese patent medicine ‘Bi Yuan Tong Qiao Granules’ to treat OSA. After 1 month of continuous medication, the apnea hypopnea index, Epworth Sleepiness Scale scores, and Fatigue Scale scores of the patients significantly decreased compared to those before treatment. In addition, acupuncture therapy^[23]^ and Ba Duan Jin^[24]^ were helpful in improving OSA. The underlying mechanism of TCM in treating OSA is not yet clear; however, TCM can effectively alleviate symptoms such as nighttime snoring, frequent awakenings, and daytime fatigue, thereby enhancing the quality of life of patients.

The common symptoms of patients with PVC belong to the category of palpitations in TCM. TCM has developed over thousands of years and has accumulated rich experience in the treatment of palpitations, with proven efficacy. In this case, the patient’s palpitations and snoring were caused by phlegm and heat interfering with the heart. The TCM treatment used was the modified Huanglian Wendan Decoction. The Huanglian Wendan Decoction has the effects of heat-clearing and phlegm-resolving, heart-nourishing, and spirit-calming.^[25]^ The randomized controlled study has confirmed the efficacy and safety of the Huanglian Wendan Decoction in PVC treatment.^[26]^ This manuscript is the first report of the treatment of OSA leading to PVC using TCM. Although the mechanism by which Huanglian Wendan Decoction treatment for PVC caused by OSA is not yet clear, modern pharmacological studies have confirmed that the multiple herbs used in this case act on various aspects of PVC induced by OSA, such as regulating the autonomic nervous system and oxidative stress, improving sleep structure, anti-myocardial ischemia, and enhancing myocardial electrical stability. The following is an overview of the common Chinese herbs.

As an effective component of Coptis chinensis, berberine fights against oxygen radicals, protects the myocardial cell membrane, prevents calcium ion overload, and blocks triggered late depolarization, thereby exerting an antiarrhythmic effect.^[27]^ Berberine improves the blood supply to ischemic areas of the myocardium by dilating coronary blood vessels and increasing coronary blood flow.^[28]^

Low doses of Agrimonia pilosa ledeb extract exert antiarrhythmic effects by regulating the synthesis and release of nitric oxide in vascular endothelial cells.^[29]^

Spikenard improves sleep structure by shortening sleep latency and extending total sleep time.^[30]^ Spikenard ethyl acetate extract increases adenosine triphosphate levels and adenosine triphosphatease activity, alleviating energy metabolism disorders caused by hypoxia, thereby providing protective effects on myocardial cells under hypoxic conditions.^[31]^

Astragalus polysaccharides significantly improves lung ventilation and increase blood oxygen content by reducing the infiltration of inflammatory cells.^[32]^ The total flavonoids of Astragalus inhibited sodium ion influx, increased the depolarization threshold of fast-response cells, reduced automaticity, and inhibited the amplitude of the potassium ion current, prolonging the action potential repolarization time, thereby exerting an antiarrhythmic effect.^[33]^

The Corydalis tuber has a significant effect on sleep phase regulation, reducing rapid eye movement sleep and deep slow-wave sleep while increasing light slow-wave sleep, thus altering sleep structure.^[34]^

Suanzaoren saponin A acts as a calcium ion channel blocker, exerting an anti-tachyarrhythmic effect by influencing L-type calcium channels in individual ventricular myocytes.^[35]^

Modern pharmacological studies of the aforementioned Chinese herbs provide a contemporary basis for the favorable outcomes observed in this patient, and this case offers insights into the treatment of PVC induced by OSA using TCM.

## 4. Conclusion

Here, we report a case of PVC induced by OSA that was treated with TCM. Given the low diagnostic rate of OSA and lack of attention, there are relatively few reports on the treatment of PVC induced by OSA. This case enriches the perspective on the treatment of PVC caused by OSA and promotes TCM as the preferred treatment strategy. In the future, more high-quality evidence is required to confirm the effects of TCM.

## Acknowledgments

Thanks to the patient for his help with this report.

## Author contributions

**Conceptualization:** Qing-Qing Yue.

**Data curation:** Qing-Qing Yue, Sheng-Yao Li, Ting Li.

**Formal analysis:** Yi-Fang Hao.

**Investigation:** Qing-Qing Yue, Yi-Fang Hao, Han-Ying Miao.

**Supervision:** Sheng-Yao Li.

**Validation:** Sheng-Yao Li, Ting Li.

**Visualization:** Sheng-Yao Li.

**Writing – original draft:** Qing-Qing Yue.

**Writing – review & editing:** Sheng-Yao Li, Han-Ying Miao, Bing-Chang Chen.
